# The Salivary Microbiome in Polycystic Ovary Syndrome (PCOS) and Its Association with Disease-Related Parameters: A Pilot Study

**DOI:** 10.3389/fmicb.2016.01270

**Published:** 2016-08-25

**Authors:** Lisa Lindheim, Mina Bashir, Julia Münzker, Christian Trummer, Verena Zachhuber, Thomas R. Pieber, Gregor Gorkiewicz, Barbara Obermayer-Pietsch

**Affiliations:** ^1^Division of Endocrinology and Diabetology, Department of Internal Medicine, Medical University GrazGraz, Austria; ^2^Center for Biomarker Research in MedicineGraz, Austria; ^3^Institute of Pathology, Medical University GrazGraz, Austria; ^4^BioTechMed, Interuniversity CooperationGraz, Austria

**Keywords:** polycystic ovary syndrome, sex steroids, human oral microbiome, next-generation sequencing, 16S rRNA, obesity, inflammation

## Abstract

**Background:** Polycystic ovary syndrome (PCOS) is a common female endocrine condition of unclear etiology characterized by hyperandrogenism, oligo/amenorrhoea, and polycystic ovarian morphology. PCOS is often complicated by infertility, overweight/obesity, insulin resistance, and low-grade inflammation. The gut microbiome is known to contribute to several of these conditions. Recently, an association between stool and saliva microbiome community profiles was shown, making saliva a possible convenient, non-invasive sample type for detecting gut microbiome changes in systemic disease. In this study, we describe the saliva microbiome of PCOS patients and the association of microbiome features with PCOS-related parameters.

**Methods:** 16S rRNA gene amplicon sequencing was performed on saliva samples from 24 PCOS patients and 20 healthy controls. Data processing and microbiome analyses were conducted in mothur and QIIME. All study subjects were characterized regarding reproductive, metabolic, and inflammatory parameters.

**Results:** PCOS patients showed a decrease in bacteria from the phylum Actinobacteria and a borderline significant shift in bacterial community composition in unweighted UniFrac analysis. No differences between patients and controls were found in alpha diversity, weighted UniFrac analysis, or on other taxonomic levels. We found no association of saliva alpha diversity, beta diversity, or taxonomic composition with serum testosterone, oligo/amenorrhoea, overweight, insulin resistance, inflammatory markers, age, or diet.

**Conclusions:** In this pilot study, patients with PCOS showed a reduced salivary relative abundance of Actinobacteria. Reproductive and metabolic components of the syndrome were not associated with saliva microbiome parameters, indicating that the majority of between-subject variation in saliva microbiome profiles remains to be explained.

## Introduction

Polycystic ovary syndrome (PCOS) is a common female endocrine condition affecting 6–18% of reproductive-age women and comprising the three primary symptoms hyperandrogenism, oligo/amenorrhoea, and polycystic ovarian morphology (Diamanti-Kandarakis et al., 1999; Asuncion et al., 2000; Azziz et al., 2004; March et al., 2010). In addition to reduced fertility, pregnancy complications, and cosmetic problems, women with PCOS are at risk to develop disorders of glucose and lipid metabolism, chronic low-grade inflammation, and the associated long-term complications (Barry et al., 2011; Escobar-Morreale et al., 2011; Lerchbaum et al., 2011, 2013; Wehr et al., 2011; Dumesic et al., 2015; Kollmann et al., 2015). Different criteria for the diagnosis of PCOS exist, leading to a wide variety of phenotypes ranging from mild to severe. Currently, the Endocrine Society recommends the use of the Rotterdam Criteria for the diagnosis of PCOS (Legro et al., 2013).

The etiology of PCOS is still unclear, although a multifactorial pathogenesis including genetic, lifestyle, and intrauterine factors has been suggested (Dumesic et al., 2015). Recent research in rodents and humans has implicated the gut microbiome in the pathogenesis of numerous diseases, including obesity, insulin resistance, and type 2 diabetes (Bäckhed et al., 2004; Turnbaugh et al., 2006, 2009; Vrieze et al., 2012). The majority of gut microbiome studies have investigated the distal digestive tract (e.g., fecal or cecal samples); however, cooling, transport, and DNA extraction methods from these sample types are non-standardized and known to cause substantial variation in sequencing results (Goodrich et al., 2014).

We have investigated the microbiome of the proximal digestive tract as a possible indicator of disease in PCOS. Saliva offers several advantages over stool as a sample material for microbiome studies. These are the non-invasive on-site collection with little or no discomfort to the patient, the possibility for immediate processing and/or freezing following collection to conserve bacterial community structures, and the use of defined, reproducible sample volumes for DNA extraction. It has recently been shown that saliva microbiome profiles correlate with those in the stool, despite the fact that the bacterial communities in the two locations differ greatly (Ding and Schloss, 2014). Therefore, saliva may be a useful alternative to stool as an indicator of bacterial dysbiosis in systemic disease.

To our knowledge, there are no published studies of either the fecal or saliva microbiome in patients with PCOS using a next-generation sequencing approach. PCOS patients exhibit an increased prevalence of gingivitis, which was found to be accompanied by changes in certain oral bacterial species, assessed by qPCR (Akcali et al., 2014). However, data on the global saliva microbiome in periodontally healthy PCOS patients compared to control women is lacking.

We performed a pilot study to describe the salivary microbiome in PCOS and to investigate the potential of specific taxa and measures of bacterial diversity to distinguish between women with PCOS and healthy women. Additionally, we investigated the association of diagnostic (serum testosterone, oligo/amenorrhoea) and common co-occurring (overweight, insulin resistance, inflammation) features of PCOS with saliva microbiome parameters. Finally, we addressed the role of age and diet as possible confounding factors in saliva microbiome studies.

## Materials and methods

### Study cohort

Twenty-five women with PCOS and 25 hormonally healthy controls were recruited from the endocrinological outpatient clinic at the University Hospital Graz. PCOS was diagnosed according to the Rotterdam Criteria, requiring the presence of two out of three of the following criteria: clinical/biochemical hyperandrogenism, oligo-/anovulation, and polycystic ovaries (Rotterdam ESHRE/ASRM-Sponsored PCOS consensus Workshop Group, 2004). Clinical hyperandrogenism, based on the presence of hirsutism, was defined as a score of eight or higher in the modified Ferriman-Gallwey (FG) assessment (Yildiz et al., 2010). Biochemical hyperandrogenism was defined as above-normal values of one or several serum androgens. Oligo-/anovulation was defined as prolonged menstrual cycles (>35 days) or the absence of menstruation for at least 3 months. Polycystic ovarian morphology in a gynecological ultrasound was assessed based on medical history. Thyroid disorder, congenital adrenal hyperplasia, Cushing's syndrome, hyperprolactinemia, androgen-secreting tumors, and pregnancy were excluded by appropriate laboratory tests and clinical examination. Healthy controls did not meet any of the Rotterdam Criteria, with the following exceptions: isolated elevation of dehydroepiandrosterone sulfate (DHEAS) or androstenedione without other signs of PCOS (6 subjects) and long-standing hirsutism without hyperandrogenemia (1 subject). Exclusion criteria for both groups were pregnancy/lactation, menopause, use of antibiotics, hormonal contraceptives, or antidiabetic medication within the preceding 3 months, gastrointestinal or periodontal disease, active infections of any kind, a body mass index (BMI) < 18, and smoking. All study participants were at least 18 years old and provided written informed consent. The study protocol was approved by the Ethics Committee at the Medical University Graz.

### Sampling

Study visits took place in the morning after an overnight fast. Study subjects were instructed not to brush their teeth and to drink only water prior to saliva sampling. Saliva was collected in the mouth for several minutes and then voided into Sali-Tubes (DRG Diagnostics, Marburg, Germany). This process was repeated until the desired volume of 1–2 ml was reached. Saliva samples were immediately cooled on ice, flash-frozen in liquid nitrogen, and stored at −70°C until further processing.

Anthropometric data were recorded and a baseline hormonal and metabolic assessment performed. Following the baseline blood sampling, a 2-h, 75 g oral glucose tolerance test (oGTT; Glucoral 75 Citron, Germania Pharmazeutika, Vienna, Austria) was performed and glucose and insulin were measured after 30, 60, and 120 min.

### Laboratory measurements

Estrone (E1), 17-estradiol (E2), total testosterone, androstenedione, dehydroepiandrosterone (DHEA), DHEAS, and dihydrotestosterone (DHT) were measured by liquid chromatography-tandem mass spectrometry at the Department of Clinical Chemistry at the University Hospital of South Manchester, Manchester, United Kingdom, as described by Keevil et al. (Chadwick et al., 2005; Owen et al., 2014, 2015; Münzker et al., 2015).

Insulin was measured by chemiluminescence immunoassay on the ADVIA Centaur XP (Roche, Rotkreuz, Switzerland). Anti-Muellerian hormone (AMH) was measured by chemiluminescence immunoassay on the Access2 (Beckman Coulter, Brea, USA). Luteinizing hormone (LH) and follicle-stimulating hormone (FSH) were measured by ELISA (both DiaSource, Louvain-la-Neuve, Belgium). Sex hormone-binding globulin (SHBG) was measured by chemiluminescence immunoassay on the Cobas e411 (Roche). Total cholesterol, high-density lipoprotein-cholesterol (HDL), triglycerides, and glucose were measured by enzymatic colorimetric assay on the Cobas c module (Roche). Serum high-sensitivity C-reactive protein (hs-CRP) was measured by ELISA (BioVendor, Brno, Czech Republic). A total and differential blood count was performed on the XE-5000 Hematology Analyzer (Sysmex, Vienna, Austria).

### Calculations and definition of terms

BMI was calculated as $\frac{\text{Weight }\left(\text{kg}\right)}{{\left(\text{Height }\left(\text{m}\right)\right)}^{2}}$. Overweight was defined as a BMI ≥ 25. The homeostasis model assessment for insulin resistance (HOMA2-IR) index was calculated using the open-source software HOMA calculator V2.2.3 provided by the Diabetes Trial Unit, University of Oxford, UK (www.dtu.ox.ac.uk/homacalculator/, last accessed Dec 17, 2015). Insulin resistance was defined as a HOMA2-IR ≥ 2. The area under the curve (AUC) for glucose and insulin was calculated from the oGTT using the trapezoidal method in GraphPad Prism 5. Free androgen index (FAI) was calculated according to the formula $100\times \frac{\text{Total testosterone }\left(\text{nmol}/\text{l}\right)}{\text{SHBG }\left(\text{nmol}/\text{l}\right)}$. Free testosterone and free DHT were calculated from total testosterone/DHT and SHBG according to Mazer et al. assuming a blood albumin concentration of 6.2 μmol/l (Mazer, 2009).

A food frequency questionnaire designed by dieticians of the Clinical Medical Nutrition Therapy Unit, University Clinic Graz, was administered to assess the intake of major food groups. Based on the results of the questionnaire, study participants were categorized as consuming a high carbohydrate or high animal protein diet.

### Next-generation sequencing

Total DNA was extracted from saliva samples using the MagNAPure LC DNA Isolation Kit III (Bacteria, Fungi) on the MagNA Pure Instrument (Roche, Rotkreuz, Switzerland). Saliva was thawed, vortexed, and 250 μl saliva was added to 250 μl bacteria lysis buffer in a sample tube containing MagNALyser Green Beads (1.4 mm diameter ceramic beads, Roche). Samples were homogenized in a MagNALyser Instrument (2 × 6000 rpm for 30 s, separated by 1 min cooling), treated with 25 μl lysozyme (Roth, Karlsruhe, Germany) at 37°C for 30 min, and then with 43.3 μl proteinase K (Roche) at 60°C for 1 h. Lysates were incubated at 95°C for 10 min, cooled on ice for 5 min, and centrifuged for 5 min at full speed. DNA was isolated from 200 μl lysate supernatant by the MagNAPure Instrument using the manufacturer's software and eluted in 100 μl elution buffer. A PCR reaction was performed to amplify the V1-2 region of the bacterial 16S rRNA gene using the primers F27 (AGAGTTTGATCCTGGCTCAG) and R357 (CTGCTGCCTYCCGTA; Eurofins Genomics, Ebersberg, Germany) and the FastStart High Fidelity PCR System, dNTPack (Roche) with initial denaturation at 95°C for 3 min followed by 28 cycles of denaturation at 95°C for 45 s, annealing at 55°C for 45 s, and extension at 72°C for 1 min, one cycle of final extension at 72°C for 7 min, and a final cooling step to 10°C. Triplicates were pooled, checked on a 1% agarose gel, and 15 μl of pooled PCR product were normalized according to manufacturer's instructions on a SequalPrep Normalization Plate (Life Technologies, Vienna, Austria). Fifteen microliters of the normalized PCR product were used as template for indexing PCR in a 50 μl single reaction to introduce barcode sequences to each sample according to Kozich et al. (2013). Cycling conditions were initial denaturation at 95°C for 3 min followed by eight cycles of denaturation at 95°C for 45 s, annealing at 55°C for 45 s, and extension at 72°C for 1 min, one cycle of final extension at 72°C for 7 min, and a final cooling step to 4°C. After indexing, 5 μl of each sample were pooled and 50 μl of the unpurified library were loaded on a 1% agarose gel and purified from the gel with the Qiaquick Gel Extraction Kit (Qiagen, Hilden, Germany) according to manufacturer's instructions. The pool was quantified using QuantiFluor ONE dsDNA dye on a Quantus Fluorometer (Promega, Mannheim, Germany) according to manufacturer's instructions and visualized for size validation on a 2100 Bioanalyzer Instrument (Agilent Technologies, Santa Clara, USA) using a high sensitivity DNA assay according to manufacturer's instructions. The final 6 pM library containing all pooled samples was run with 20% PhiX and version 3, 600 cycles chemistry according to manufacturer's instructions on a MiSeq desktop sequencer (Illumina, Eindhoven, Netherlands). One negative control (250 μl sterile PBS instead of saliva) was included in each MagNAPure run and subjected to the same procedures as samples. A mock community containing genomic DNA from 20 bacterial species was included to estimate PCR and sequencing errors (HM-782D, BEI Resources, Manassas, USA).

### Sequencing data analysis

Raw reads were processed using the open-source software mothur V1.35.0 according to the protocol by Kozich et al. (April 2015), with the following adaptations: no maxlength was defined during the screening step, start (1046) and end (6426) positions were adapted to the V1-V2 region, and a difference of 3 bases was permitted during the precluster step (based on the recommendation by the authors to allow one mismatch per 100 bp; Kozich et al., 2013). Chimeric sequences were removed by UCHIME (Edgar et al., 2011). After removal of non-bacterial sequences, classified using the SILVA119 database (www.arb-silva.de), the remaining sequences were degapped, deuniqued, split into individual samples, and formatted for use with the open-source software QIIME 1.8.0 (Caporaso et al., 2010). Open reference operational taxonomic unit (OTU) picking was performed in QIIME using UCLUST against the Greengenes 13.8 database (DeSantis et al., 2006). An OTU was defined as a group of sequences with a similarity of 97% or more. Based on the mock community sequencing results, a relative abundance cutoff of 0.1% was applied for subsequent analyses. Faith's phylogenetic diversity and the number of observed OTUs were used as metrics for alpha rarefaction, which was performed in QIIME. Principal coordinate analyses (PCoA) were based on unweighted and weighted UniFrac distances and calculated in QIIME (Lozupone and Knight, 2005). Taxa summaries were performed in QIIME. All samples were normalized to the sample with the lowest read count for alpha and beta diversity comparisons. For taxa comparisons, relative abundances based on all obtained reads were used.

Raw sequencing data are available in NCBI's short read archive (SRA) under the accession number SRP077213.

### Statistical analysis

Nonparametric student's *t*-tests using Monte Carlo permutations were used for alpha diversity comparisons, Mann–Whitney *U*-tests for taxa comparisons, and Adonis for category comparisons of distance matrices, all calculated in QIIME. Benjamini–Hochberg false discovery rate (FDR) correction was used to correct for multiple hypothesis testing where applicable.

All remaining statistical calculations were performed in IBM SPSS Statistics Version 22. Depending on the statistical distribution of the variable, unpaired *t*-tests or Mann–Whitney *U*-tests were used to compare groups. Fisher's Exact tests were used to compare categorical parameters. All data are expressed as median and interquartile range (IQR).

## Results

### Study subject characteristics

All 50 subjects included in the study provided saliva samples. Three subjects were excluded from the control group due to previously undetected hyperandrogenemia (elevation of two or more androgens in fasted blood sample), two subjects were excluded due to smoking prior to sampling, and one subject was excluded due to a BMI < 18. The final analyses were performed with 20 healthy controls and 24 PCOS patients.

Laboratory, anthropometric, and patient history data are summarized in Table 1. Patients with PCOS had significantly higher total testosterone, androstenedione, and DHEA (*p* = 0.002, <0.001, and 0.015, respectively) and lower E2 (*p* < 0.001) levels than healthy controls, while no difference was found for DHEAS, DHT, and E1 (*p* = 0.073, 0.096, and 0.138, respectively). Calculated free DHT, free testosterone, and FAI were higher in the PCOS group (*p* < 0.001 for all). PCOS patients showed a characteristic dysregulation of FSH and LH secretion, with increased LH levels compared to controls (*p* = 0.035). Hirsutism and oligo/anmenorrhoea were more prevalent in the PCOS group (*p* = 0.003 and <0.001, respectively). Nearly all PCOS patients reported a history of polycystic ovaries (*p* < 0.001), which was corroborated by elevated AMH levels at the time of sampling (*p* < 0.001). An increased basal insulin secretion and AUCinsulin in the oGTT, elevated total triglycerides, and reduced HDL-cholesterol were observed in the PCOS group (*p* = 0.022, 0.009, 0.010, and 0.006, respectively). The studied cohort included lean as well as obese PCOS patients. Overall, BMI did not differ between PCOS patients and controls (*p* = 0.147). Total blood leukocytes were significantly higher in PCOS patients compared to healthy controls (*p* = 0.040), while hsCRP was not significantly different between the two groups (*p* = 0.078).

**Table 1:** **Study subject characteristics**.

	**Reference Range**	**Control (*n* = 20)**		**PCOS (*n* = 24)**		
		**Median**	**IQR**	**Median**	**IQR**	***p*-value**
Age		32	12.0	27	5.9	0.003^**^
Body mass index	18.5–25.0^#^	22.3	4.10	24.9	11.75	0.147
Waist to hip ratio	<0.85^#^	0.80	0.063	0.82	0.077	0.439
Fasting glucose (mmol/l)	<7.0^†^	4.5	0.50	4.7	0.59	0.209
2h glucose (mmol/l)	<11.1^†^	4.3	1.09	4.8	1.15	0.296
AUC glucose (mmolh/l)	^§^	10.2	4.52	10.9	3.61	0.273
Fasting insulin (pmol/l)	20.9–173.8	41.4	51.08	84.4	55.25	0.022^*^
2h insulin (pmol/l)	^§^	129	140.0	188	336.7	0.371
AUC insulin (mmolh/l)	^§^	353	427.3	691	562.0	0.009^**^
HOMA2-IR	<2	0.8	1.05	1.7	1.20	0.027^*^
Total cholesterol (mmol/l)	<5.2	4.6	0.64	4.5	1.13	0.699
HDL-cholesterol (mmol/l)	>1.0	2.0	0.42	1.7	0.49	0.006^**^
Triglycerides (mmol/l)	<1.65	0.59	0.248	0.74	0.242	0.010^*^
Follicle-stimulating hormone (IU/l)	0.5–61.2^‡^	9.2	8.11	7.5	2.73	0.178
Luteinizing hormone (IU/l)	2.0–22.0^‡^	5.8	9.34	9.3	8.60	0.042^*^
LH:FSH ratio	^§^	1.2	1.19	1.5	1.06	0.035^*^
Anti-Muellerian hormone (pmol/l)	1.4–65.2	26.8	22.42	61.1	52.59	<0.001^***^
Total testosterone (nmol/l)	0.37–2.12	1.1	0.56	1.3	0.77	0.002^**^
Dihydrotestosterone (nmol/l)	^§^	0.34	0.241	0.46	0.528	0.096
Androstenedione (nmol/l)	0.89–7.46	2.6	1.61	4.2	2.69	<0.001^***^
Dehydroepiandrosterone (nmol/l)	^§^	13.7	11.37	21.4	12.40	0.015^*^
Dehydroepiandrosterone sulfate (μmol/l)	^§^	3.3	3.74	4.9	2.35	0.073
Estrone (pmol/l)	^§^	274	184.8	195	118.9	0.138
17-Estradiol (pmol/l)	^§^	436	285.8	163	181.1	<0.001^***^
Free androgen index	^§^	1.3	0.68	3.1	2.75	<0.001^***^
Free testosterone (pmol/l)	^§^	10.6	5.86	20.9	13.00	<0.001^***^
Free dihydrotestosterone (pmol/l)	^§^	1.3	1.03	3.0	2.19	<0.001^***^
Total blood leukocytes (G/l)	4.4–11.3	4.7	1.47	5.5	1.78	0.040^*^
hsCRP (mg/l)	^§^	0.5	0.70	0.8	3.97	0.078
		**# of cases**	**% of cases**	**# of cases**	**% of cases**	***p*****-value**
Polycystic ovarian morphology		0	0	22	96	<0.001^***^
Hirsutism		1	5	11	46	0.003^**^
Oligo-/Amenorrhoea		1	5	17	71	<0.001^***^
High carbohydrate diet		8	40	9	38	0.555
High animal protein diet		12	60	15	63	0.555

*PCOS, polycystic ovary syndrome; IQR, interquartile range; AUC, area under the curve; HOMA2-IR, homeostasis model assessment for insulin resistance; HDL, high density lipoprotein; hsCRP, high-sensitivity C-reactive protein. Groups were compared using unpaired t-tests, Mann–Whitney U-tests, and Fisher's Exact tests*.

# *according to the World Health Organization*,

† *according to the American Diabetes Association*,

‡ *depending on menstrual cycle stage*,

§ *reference range not available*.

* *p < 0.05*,

** *p < 0.01*,

*** *p < 0.001*.

### Assessment of sequencing error and bias using a mock community

A mock community containing genomic DNA from twenty bacterial species, representing 17 genera, was included in the 16S rRNA PCR and sequencing to estimate OTU inflation and classification bias due to sequencing errors. After removal of singleton OTUs, we detected 214 OTUs from 29 genera in the mock community sample, indicating an overestimation of the number of OTUs due to sequencing errors (Table 2). After filtering the mock community and our dataset to 1, 0.1, and 0.01% relative abundance, we determined that a cutoff of 0.1% best represented the mock community, detecting 31 OTUs from 19 genera (Supplementary Data Sheet 1). We therefore performed the subsequent analysis using this abundance filter.

**Table 2:** **Expected and observed relative abundances of bacterial genera in a mock community**.

**Genus**	**Expected RA**	**Observed RA**	**Fold Δ**
^#^Acinetobacter	0.05	0.02	−2.4
^†^Actinomyces	0.05	0.03	−1.6
^‡^Bacillus	0.05	0.04	−1.2
^§^ Bacteroides	0.05	0.12	2.4
^‡^Clostridiaceae unclass	0.05	0.06	1.2
^||^Deinococcus	0.05	0.04	−1.2
^‡^Enterococcaceae unclass	0.05	0.04	−1.4
^#^Enterobacteriaceae unclass	0.05	0.02	−2.2
^#^Helicobacter	0.05	0.11	2.2
^‡^Lactobacillus	0.05	0.06	1.3
^‡^Listeria	0.05	0.05	1.0
^#^Neisseria	0.05	0.06	1.3
^†^Proprionibacterium	0.05	0.06	1.1
^#^Pseudomonas	0.05	0.02	−2.7
^#^Rhodobacter	0.05	0.04	−1.2
^‡^Staphylococcus (2 spp.)	0.10	0.09	−1.2
^‡^Streptococcus (3 spp.)	0.15	0.13	−1.1

*Only sequences with a relative abundance >0.1% were included in the analysis*.

# *Proteobacteria*,

† *Actinobacteria*,

‡ *Firmicutes*,

§ *Bacteroidetes*,

|| *[Thermi]. RA, relative abundance; unclass., unclassified; spp., species*.

Using the 0.1% cutoff, all bacteria in the mock community were correctly classified at the family level, 15/17 at the genus level, and 7/20 at the species level (Supplementary Data Sheet 1). The observed relative abundance of most genera was within 50% of the expected value (Table 2). Bacteria from the genera Bacteroides and Helicobacter were more than two-fold overestimated, while bacteria from the family Gammaproteobacteria were more than two-fold underestimated (Table 2).

### The saliva microbiome composition in PCOS and its association with metabolic dysfunction and inflammation

16S rRNA amplicon-based microbiome analysis was performed on saliva samples from 20 healthy controls and 24 PCOS patients, using an OTU relative abundance cutoff of 0.1%. A median of 80,555 (IQR 18,509) and 72,284 (IQR 20,330) paired-end Illumina reads were analyzed per sample in the control and PCOS groups, respectively (*p* = 0.131). A total number of 131 OTUs [median(IQR) = 119.5(9.0) for controls and 116(8.5) for PCOS] from 35 genera [median(IQR) = 33(1.0) for controls and PCOS] were identified. As PCOS is often accompanied by overweight/obesity, insulin resistance, and chronic low-grade inflammation, we investigated the association of these features with saliva microbiome profiles. In addition, we performed analysis with samples grouped by diet and age, as these factors have been shown to influence gut microbiome composition (33–35).

The saliva microbiome was dominated by bacteria from the phylum Bacteroidetes (median relative abundance 45%) and Firmicutes (26%), while bacteria from the phyla Proteobacteria, Fusobacteria, Actinobacteria, and TM7 contributed <10% each to total bacterial content (Table 3). On genus level, Prevotella was the single most abundant genus (median relative abundance 31%), followed by Streptococcus (11%), with other genera contributing <10% each to total bacterial content (Table 3). Saliva samples from PCOS patients showed a significant decrease in the relative abundance of bacteria from the phylum Actinobacteria (FDR *p* = 0.024). On class, order, family, genus, and OTU level, no differences were observed between patients and controls (Table 3, Supplementary Data Sheet 2).

**Table 3:** **Relative abundances of bacterial genera and phyla with a median relative abundance >1%**.

**Most abundant genera (>1%)**	**% of total bacteria in Control (*n* = 20)**		**% of total bacteria in PCOS (*n* = 24)**		**FDR *p*-value**
	**Median**	**IQR**	**Median**	**IQR**	
^#^Prevotella	32.5	8.63	30.8	4.17	0.981
^†^Streptococcus	10.3	2.69	12.2	8.29	0.740
^†^Veillonella	8.4	3.65	8.2	5.42	0.847
^#^[Prevotella]	7.1	7.67	6.5	5.75	0.740
^‡^Neisseria	6.9	5.74	5.5	7.63	0.740
^§^Fusobacterium	3.8	3.44	5.4	4.11	0.749
^#^Porphyromonas	3.0	5.63	5.5	4.60	0.749
^||^Rothia	4.5	3.31	2.7	2.33	0.726
^‡^Haemophilus	3.0	2.06	2.3	1.81	0.910
^§^Leptotrichia	2.1	4.00	1.5	2.25	0.740
^||^Actinomyces	2.4	0.93	2.3	1.30	0.749
^†^Granulicatella	1.5	1.04	1.4	0.83	0.740
^||^Atopobium	1.0	1.11	0.7	0.61	0.740
^†^Gemellaceae unclass	0.9	0.76	0.7	0.69	0.941
^‡^Campylobacter	0.8	0.50	0.7	0.53	0.749
^¶^TM7-3 unclass	0.8	0.78	0.6	0.75	0.740
**Most abundant phyla (**>**1%)**	**Median**	**IQR**	**Median**	**IQR**	**FDR** ***P*****-value**
^#^Bacteroidetes	43.9	6.70	46.4	6.88	0.706
^†^Firmicutes	24.8	6.21	27.1	7.98	0.706
^‡^Proteobacteria	9.5	8.94	10.1	8.21	0.706
^§^Fusobacteria	7.2	4.58	7.3	5.37	0.706
^||^Actinobacteria	8.2	2.19	6.1	2.82	0.024^*^
^¶^TM7	1.3	1.38	1.1	1.11	0.492

*Groups were compared using Mann–Whitney U-tests followed by Benjamini-Hochberg FDR correction. PCOS, polycystic ovary syndrome; IQR, interquartile range*.

# *Bacteroidetes*,

† *Firmicutes*,

‡ *Proteobacteria*,

§ *Fusobacteria*,

|| *Actinobacteria*,

¶ *TM7. Square brackets indicate a Greengenes suggested taxonomic assignment*.

* *p < 0.05*.

**Figure 1:**
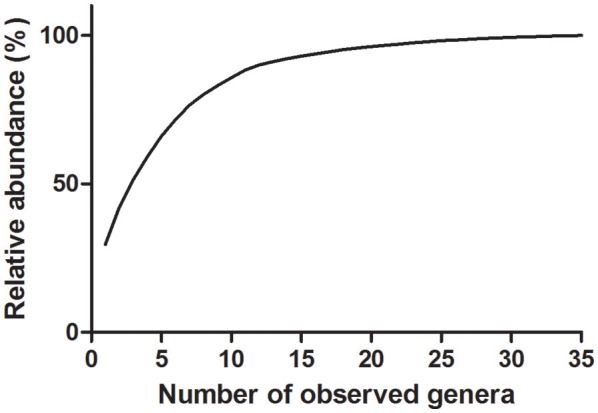
**Cumulative distribution of genus relative abundances in saliva samples**. Thirty-five genera were detected in saliva samples. Eighty-six percent of all identified bacteria are represented by the 10 most abundant genera.

The cumulative curve of observed genus level abundances followed a long-tailed distribution, with the ten most abundant genera accounting for 86% of all identified bacteria (Figure 1).

**Figure 2:**
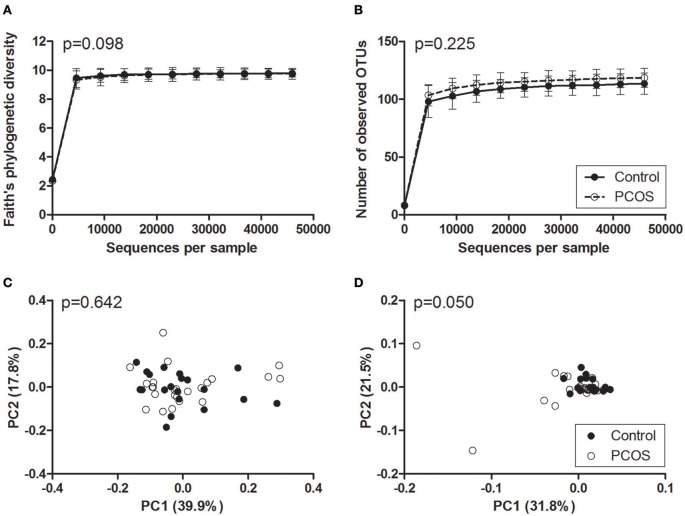
**Alpha and beta diversity of saliva samples from PCOS patients and controls. (A,B)** Alpha rarefaction curves of Faith's phylogenetic diversity **(A)** and the number of observed OTUs **(B)**. Samples were rarefied to the smallest observed number of reads (45,949). Median and IQR are plotted. **(C,D)** Principal coordinate analysis (PCoA) plots of weighted **(C)** and unweighted **(D)** UniFrac distances. Each dot represents the bacterial community composition of one individual saliva sample. Groups were compared using Monte Carlo permutations for alpha diversity and Adonis for beta diversity. PCOS, polycystic ovary syndrome.

**Figure 3:**
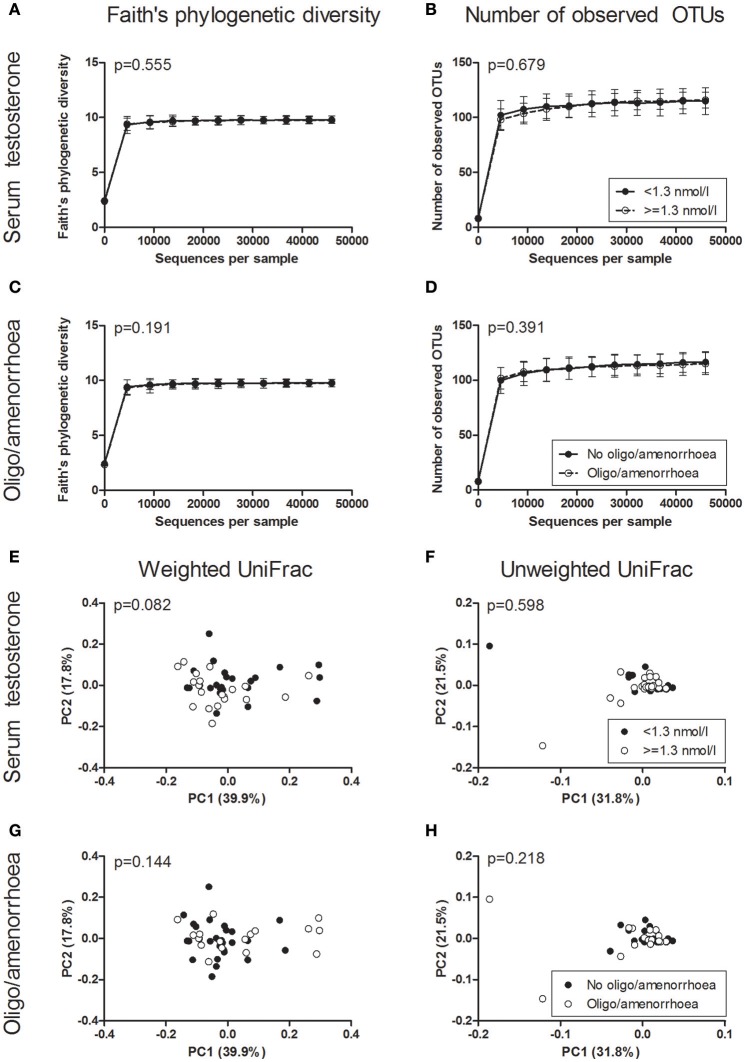
**Alpha and beta diversity of saliva samples based on PCOS diagnostic criteria. (A–D)** Alpha rarefaction curves of Faith's phylogenetic diversity **(A,C)** and the number of observed OTUs **(B,D)** grouped by serum testosterone **(A,B)** and the presence of oligo/amenorrhoea **(C,D)**. Samples were rarefied to the smallest observed number of reads (45,949). Median and IQR are plotted. **(E–H)** Principal coordinate analysis (PCoA) plots of weighted **(E,G)** and unweighted **(F,H)** UniFrac distances grouped by serum testosterone **(E,F)** and the presence of oligo/amenorrhoea **(G,H)**. Each dot represents the bacterial community composition of one individual saliva sample. Groups were compared using Monte Carlo permutations for alpha diversity and Adonis for beta diversity. PCOS, polycystic ovary syndrome.

Faith's phylogenetic diversity and the number of observed OTUs did not differ between PCOS patients and controls (Figure 2). Additionally, serum testosterone and the presence of oligo-/amenorrhoea were not associated with a change in these parameters (Figure 3). Alpha diversity curves of individual samples showed excellent saturation both unrarefied and at the selected rarefaction level of 45,949 reads (Supplementary Image 1).

In beta diversity analyses, saliva samples showed a tendency toward a statistically significant clustering in unweighted UniFrac analysis (Figure 2D, *p* = 0.050). No clustering was observed when comparing weighted UniFrac distance matrices based on PCOS status, serum testosterone, or the presence of oligo-/amenorrhoea (Figures 2C, 3).

Grouping samples based on overweight, insulin resistance, hsCRP, blood leukocytes, age, and diet did not affect alpha diversity, beta diversity, or taxonomic composition (data not shown).

## Discussion

To our knowledge, this is the first study reporting a next-generation sequencing-based profile of the saliva microbiome in PCOS patients. The phyla and genera that were found to dominate saliva microbiome profiles in our study cohort correspond to those previously reported for healthy adults (Keijser et al., 2008; De Filippis et al., 2014; Ding and Schloss, 2014). We show that PCOS is associated with a decreased relative abundance of salivary Actinobacteria and a borderline significant clustering of bacterial profiles in unweighted UniFrac analysis. This observation was not explained by individual components of the syndrome, namely hyperandrogenemia and oligo/amenorrhoea, or by associated features such as overweight, insulin resistance, and low-grade inflammation.

Actinobacteria, a phylum of gram-positive bacteria, are common members of the skin and oral microbiota and have been reported to be reduced in periodontal disease (Liu et al., 2012; Wang et al., 2013). Akcali et al. used quantitative real-time polymerase chain reaction to show changes in several bacterial species in women with PCOS and gingivitis compared to healthy women with gingivitis (Akcali et al., 2014). However, the authors observed no change between periodontally healthy women with and without PCOS. As our PCOS patients were periodontally healthy and we did not observe an association between the saliva microbiome and markers of inflammation, we hypothesize that the reduction in the relative abundance of Actinobacteria within the context of PCOS does not itself cause disease, but rather provides a more favorable environment for pathology-associated bacteria, which can result in periodontal disease in the presence of other permissive factors. This hypothesis is supported by the fact that the prevalence of periodontal disease is higher in PCOS patients than in the general population (Porwal et al., 2014; Rahiminejad et al., 2015).

Salivary microbiome profiles of PCOS patients showed a borderline significant clustering in unweighted UniFrac analysis, while weighted UniFrac distance matrices and alpha diversity metrics were not significantly different to controls. Several explanations exist for this apparent lack of pronounced differences. On average, the patients in our study displayed mild phenotypes of PCOS, in that serum androgens were only slightly elevated compared to controls, parameters related to glucose and lipid metabolism were within the normal range for most patients, and BMI was not significantly different from controls. We did not specifically recruit only lean or obese PCOS patients, as we were aiming for a broadly representative cohort of PCOS phenotypes. Shifts in the saliva microbiome may parallel the clinical phenotype, becoming more pronounced in the presence of severe hyperandrogenism and anovulation, either alone or in combination with obesity and/or manifest type 2 diabetes. Furthermore, the approach of this study provides only information on the presence of bacterial DNA, but not on bacterial function. Salivary bacteria may have altered gene expression patterns either in response or as a contributing factor to the biochemical changes observed in PCOS.

As it is known that the microbiome can be affected by many exogenous and endogenous factors (Goodrich et al., 2014), we addressed these possible confounders by either defining them as an exclusion criterion (such as smoking, antibiotics use, and periodontal disease) or by performing separate analyses for these variables (as for BMI, insulin resistance, inflammation, diet, and age). We did not find an association of any of these factors with alpha diversity, beta diversity, or changes in bacterial relative abundance on any taxonomic level.

Recent research has indicated that the effect of microbiome “confounders” may be less significant than previously assumed. Studies by De Filippis et al. and Belstrøm et al. have reported no effect of age and diet on saliva microbiome profiles (Belstrøm et al., 2014; De Filippis et al., 2014), while another study showed no effect of gender and BMI (Stahringer et al., 2012). Chen et al. investigated the effect of race, BMI, alcohol intake, sex, tobacco use, and age on the stool microbiome and found that each factor explained <1% of variability in stool microbiome profiles (Chen et al., 2016). These studies, together with our results, illustrate the large knowledge gaps that still exist about the factors shaping the microbiome, currently termed “inter-individual variation.”

The Illumina approach which we selected is among those with the highest sequencing depth (Sims et al., 2014). Therefore, we do not expect a great improvement of taxonomic resolution with an even higher coverage. This could be achieved by increasing the length of the sequenced 16S rRNA gene fragment. Short read lengths are a limitation of Illumina paired-end sequencing. However, lower sequencing errors compared to pyrosequencing and IonTorrent led us to prefer this approach over one employing longer read lengths but lower quality. By including a mock community containing genomic DNA from twenty bacterial species in equal concentrations, we were able to assess PCR and sequencing bias. We found that the relative abundances of the genera Bacteroides and Helicobacter were overestimated, while the relative abundance of the family Gammaproteobacteria was underestimated by our employed sequencing approach. It should be noted that the bacterial community representation of saliva samples may deviate from this pattern, as DNA extraction method is also a known source of bias which was not assessed by the mock community (Goodrich et al., 2014).

The main strength of our study is the thorough characterization of our study cohort, which included an assessment of ovarian and adrenal androgens, lipid metabolism, and glucose tolerance. Furthermore, we applied strict exclusion criteria to ensure that no subjects with an undiagnosed mild form of PCOS or other hormonal imbalance were included in the control group and to eliminate factors which may influence the saliva microbiome, such as smoking, the use of antibiotics, and periodontal disease. A second strength is our sampling approach. We collected saliva samples after an overnight fast, avoiding a disturbance of the oral microbiome due to brushing teeth or using mouthwash. Samples were immediately frozen in liquid nitrogen to optimally conserve the bacterial community structure at the time of sampling. Finally, we used a mock community to evaluate the quality of the sequencing methodology and found that a large percentage of OTUs were most likely the result of sequencing errors. By removing these OTUs, we greatly improved the validity of our results. Since we thereby also removed a proportion of true sequences, we applied several filters at different relative abundance levels, as well as performing an analysis on unfiltered data, to attain a balanced interpretation of the bacterial composition of our samples. The significant result was obtained only when using the 0.1% filter, therefore it should be interpreted with caution until it can be replicated in a larger cohort of patients.

Weaknesses of our study are the small sample size, which precluded stratification of PCOS subtypes, and the paucity of extreme phenotypes. However, this pilot study was designed to represent a spectrum of typical Austrian PCOS phenotypes, allowing a first glimpse at the saliva microbiome in this common condition. Future studies should aim to recruit large patient and control groups and stratify based on different PCOS phenotypes, either based on Rotterdam vs. NIH diagnostic criteria or as described by Jamil et al. (Dumesic et al., 2007; Jamil et al., 2015). The addition of a “positive control,” such as patients with periodontal disease, would underscore the presence or lack of differences due to PCOS alone and confirm that our result was not obtained due to technical shortcomings of the employed method.

While the saliva microbiome appears to be only minorly changed in PCOS, the microbiome of other body areas may play a more significant role in this pathology. Two research groups have recently shown an alteration in the fecal microbiome of two different rodent models of PCOS (Guo et al., 2016; Kelley et al., 2016). Furthermore, bacterial colonization of the vagina and ovarian follicles was found to affect the outcome of assisted reproductive treatment in women with infertility of various etiologies, including PCOS (Pelzer et al., 2011). Next-generation sequencing of samples from these body sites in PCOS patients presents an interesting approach for future studies.

In conclusion, we present a first report of the saliva microbiome composition in PCOS. In our cohort, PCOS patients showed a reduced relative abundance of bacteria from the Actinobacteria phylum, while bacterial community composition and diversity seems to be independent of the reproductive and metabolic abnormalities observed in these patients. Larger studies with stratification of PCOS phenotypes are needed to clarify the presence or absence of microbiome changes due to different components of the syndrome. Bacterial functionality, assessed by metagenomics and metatranscriptomics, can provide further insights into the role of salivary bacteria in this condition.

## Author contributions

LL: study design, patient recruitment, data collection, laboratory analyses, data analysis, interpretation of results, drafting of manuscript; MB: sequencing data analysis, interpretation of results; JM: coordination of steroid hormone measurements, laboratory analyses, interpretation of results; CT: patient recruitment; VZ: laboratory analyses; TP: interpretation of results; GG: study design, interpretation of results; BO: study design, interpretation of results, study supervision. All authors were involved in the revision and approved the final version of the manuscript.

## Funding

This work was funded by the DK-MOLIN (Austrian Science Fund (FWF) W1241) and the Medical University of Graz. The funding source was not involved in the study design, data collection, analysis, or interpretation, drafting of, and decision to publish the manuscript.

### Conflict of interest statement

The authors declare that the research was conducted in the absence of any commercial or financial relationships that could be construed as a potential conflict of interest.

## Supplementary material

Supplementary Data Sheet 1 **Mock community sequencing results**. Expected and observed classification and relative abundances of a mock community at genus and OTU levels using singleton removal and relative abundance cutoffs at 0.1 and 0.01%. Square brackets indicate a Greengenes suggested taxonomic assignment.

Supplementary Data Sheet 2 **Taxa comparisons of saliva samples from PCOS patients and healthy controls**. Detected taxa and group comparisons on phylum, class, order, family, genus, and OTU level. FDR, Benjamini-Hochberg false discovery rate correction for multiple testing. Mean relative abundances are shown. Square brackets indicate a Greengenes suggested taxonomic assignment.

Supplementary Image 1 **Alpha diversity of individual saliva samples**. Faith's phylogenetic diversity (**A**, PD_whole_tree) and the number of observed OTUs (**B**, observed_species) of individual saliva samples plotted against the number of reads analyzed.
