# Supplementary figures and images for: The Salivary Microbiome in Polycystic Ovary Syndrome (PCOS) and Its Association with Disease-Related Parameters: A Pilot Study
Source: Front Microbiol. 2016 Aug 25;7:1270. doi: 10.3389/fmicb.2016.01270 (PMC4996828; doi:10.3389/fmicb.2016.01270)

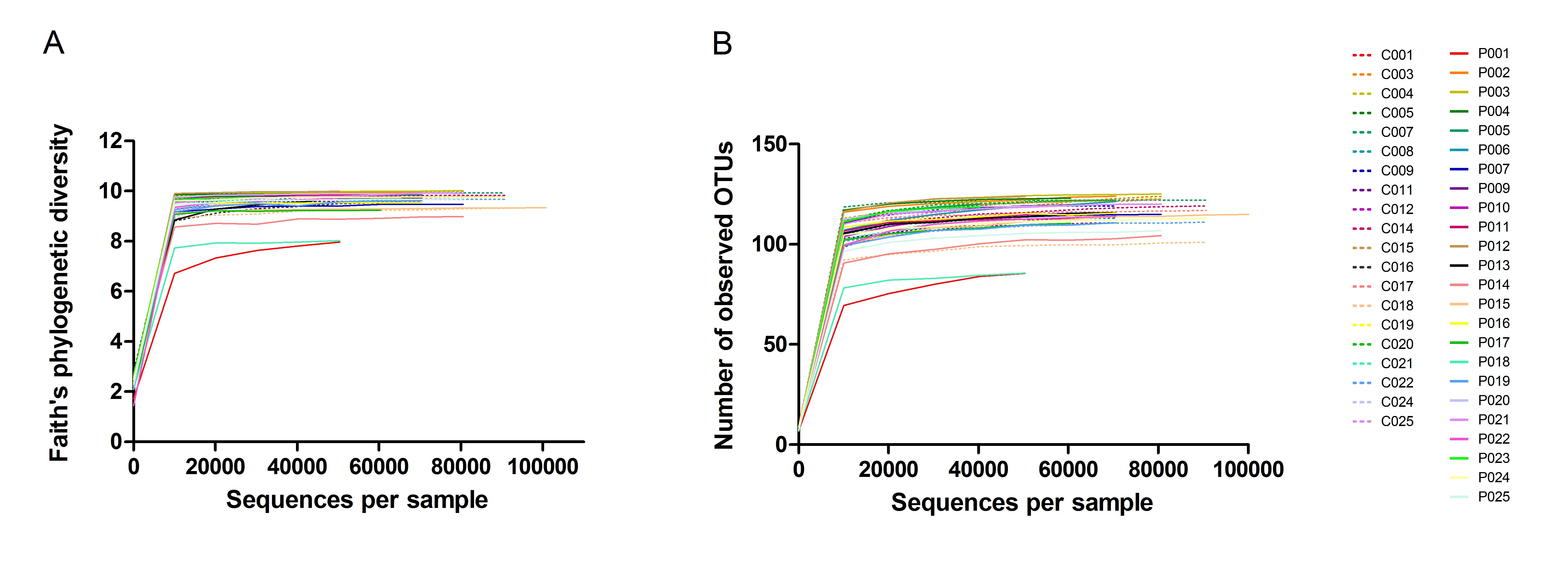

Supplement: Supplementary Image 1 — Alpha diversity of individual saliva samples. Faith's phylogenetic diversity (A, PD_whole_tree) and the number of observed OTUs (B, observed_species) of individual saliva samples plotted against the number of reads analyzed. [file Image1.PNG]
